# Precious Stones of the Month

**Published:** 1891-06

**Authors:** 


### PRECIOUS STONES OF THE MONTHS.

If each flower, in the lore that has come down to us, has a significance, so has each of the precious stones, which not only is used to symbolize mental and moral characteristics, but the month of the year, which in old Pagan days, and even yet amongst the ignorant in some countries, had a supposed bearing on personal history and traits. The following are the stones especially connected with the months of the year: January, a jacinth, or garnet, which denotes constancy and fidelity in every engagement; February, amethyst, insuring peace of mind; March, a bloodstone, denoting courage and secrecy in dangerous enterprises; April, sapphire or diamond, signifying repentance and innocence; May, the green emerald, typical of love; June, an agate, meaning long life and health. July, ruby or cornelian, which insures the forgetfulness or cure of evils springing from friendship or love. August, sardonyx, a happy married life; September, chrysolite, which preserves from folly; October, aquamarine or opal, which denotes both misfortune and hope; November, the topaz, bringing the owner fidelity and friendship; December, turquoise or malachite, signifying the most brilliant success and happiness.

